# A family navigator model for foster parents to promote youth mental health wellness: Study development and protocol

**DOI:** 10.1016/j.conctc.2026.101651

**Published:** 2026-06-06

**Authors:** Christina L. Boisseau, Heather Risser, Miguel A. Herrera, Clara Law, Zoran Martinovich, Spencer St. Jean, Yufan Wang

**Affiliations:** a Department of Psychiatry and Behavioral Sciences, Northwestern University Feinberg School of Medicine, Chicago, IL, USA; b Department of Psychology, CUNY- Hunter, NY, USA

**Keywords:** Child welfare, Foster care, Kinship care, Family navigation, Foster parent, Youth in care

## Abstract

**Background:**

Children in foster care experience disproportionately high rates of mental illness, yet most do not receive timely or evidence-based mental health treatment. Foster parents play a critical role in treatment engagement but face substantial structural and informational barriers to accessing care.

**Objective:**

This study describes the development, human-centered design, open and pilot randomized controlled trial of *Family Navigator Plus (FN+)*, a telehealth-delivered family navigation intervention designed to increase foster parent activation and reduce barriers to mental health treatment access for children in foster care.

**Methods:**

FN+ is a 5-week, manualized intervention delivered via telehealth by trained community health workers. The study includes three phases: 1) human-centered design interviews with foster parents, caseworkers, and providers; 2) an open trial (*N =* 12) to refine the intervention; and 3) a pilot randomized controlled trial (*N* = 60) comparing FN + to an attention control condition focused on youth civic engagement. The primary outcome is foster parent activation in the child's mental health care.

**Conclusions:**

This study will provide critical feasibility, acceptability, and preliminary effectiveness data for a scalable, cost-effective approach to improving mental health care access for children in foster care.

## 1 Introduction

Children involved in the child welfare system represent one of the highest-risk pediatric populations for mental health disorders in the United States. Over 300,000 youth are currently living in foster care placements [1], with approximately 20,000 youth newly entering care each year. Removal from the home is frequently preceded and followed by exposure to multiple forms of trauma, including maltreatment, caregiver loss, and placement instability [2]. As a result, children in foster care experience disproportionately high rates of depression, anxiety, and trauma-related symptoms [[3], [4], [5], [6]]. Approximately half of children in foster care exhibit clinically significant mental health symptoms, risk behaviors, or diagnosable psychiatric disorders [7]. Relative to the general pediatric population, children in foster care are more than three times as likely to attempt suicide, five times more likely to be diagnosed with anxiety, seven times more likely to be diagnosed with depression, and six times more likely to have behavioral problems [8,9].

Despite high rates of psychiatric morbidity, most children in foster care do not receive timely or adequate mental health treatment [[10], [11], [12]]. Treatment delays exceeding a decade are common [13]. When services are accessed, these youth are less likely than their peers to receive evidence-based interventions [14,15]. This gap in care is particularly concerning given that early mental health intervention can prevent the progression of psychiatric disorders and significantly reduce long-term morbidity and mortality [13,16]. Together, these disparities highlight the critical importance of ensuring timely access to appropriate, evidence-based mental health care for children in foster care to mitigate long-term adverse outcomes.

Foster parents play a central role in recognizing children's mental health needs, initiating care, and sustaining treatment engagement, yet they face numerous structural and practical barriers. In most cases, they are not authorized to independently consent for or enroll children in mental health treatment, necessitating coordination with child welfare caseworkers [7,12,17]. At the same time, many foster parents report limited training in identifying mental health symptoms, understanding treatment options, or navigating complex and fragmented healthcare systems [12,[17], [18], [19]]. These challenges are further compounded by the difficult-to-manage behaviors common among children in foster care, including emotional dysregulation, impulsivity, and self-injurious behaviors [7,20]. Such behaviors increase caregiver stress and are associated with placement disruptions, which in turn further exacerbate children's mental health difficulties [[20], [21], [22]]. Accordingly, foster parents consistently express a desire for structured support to help them navigate services, collaborate effectively with caseworkers, and understand when and how to engage mental health treatment [23,24].

Family navigation models are well suited to addressing barriers to mental health care for children in foster care. These models combine time-limited case management with care coordination to help families overcome system-level obstacles, access appropriate services, and sustain treatment engagement within complex healthcare environments [25]. Although family navigation has shown promise across pediatric populations, evidence specific to pediatric mental health remains limited. Existing studies suggest that family navigation is associated with small to moderate improvements in parent knowledge and confidence, action-taking, reduced time to treatment, satisfaction with care, and caregiver depressive symptoms [26,27].

One proposed mechanism underlying these effects is increased parental activation. Parental activation refers to a caregiver's knowledge, skills, and confidence to effectively manage a child's mental health needs and engage with care systems on the child's behalf [28]. For example, an activated foster parent may recognize early signs of distress, ask informed questions about treatment options, advocate for evaluations or services, monitor symptoms between visits, and implement therapeutic strategies at home. Unlike parental involvement, which reflects participation in care (e.g., attending appointments), or parental education, which focuses primarily on knowledge acquisition, parental activation reflects the caregiver's capacity to take effective, goal-directed action across these domains. In pediatric and adult health settings, higher parent or patient activation has been associated with greater treatment engagement, improved adherence, and better clinical outcomes. Within child welfare settings, greater foster parent activation may facilitate earlier identification of mental health needs, more effective navigation of fragmented service systems, and more consistent treatment participation, potentially reducing placement disruptions while improving caregiver competence, satisfaction, and retention.

Building on this emerging evidence, we developed the initial prototype of *Family Navigator Plus* (*FN+,* originally named *PROACTIVE parent*), a telehealth-delivered intervention designed to increase foster parent activation in managing their foster child's mental care [26]. The current NIH-funded study includes three phases designed to further refine and pilot test FN+: 1) human-centered design workshops with foster parents, caseworkers, and providers; 2) an open trial (*N* = 12) to refine the intervention; and 3) a pilot randomized controlled trial (*N* = 60) comparing FN + to an attention control condition focused on youth civic engagement. The primary outcome is foster parent activation in the child's mental health care. The secondary outcome is overcoming barriers to treatment engagement. FN + aims to equip foster parents with sustainable skills to navigate mental health systems, respond effectively to children's mental health needs, and support treatment goals over time.

## 2 Design and pilot testing

### 2.1 Phase I: Human-centered design of the FN + program curriculum

To improve user experience and acceptability of the FN + program, we conducted structured interviews or focus groups with foster parents (*n* = 6), licensed mental health providers treating children in foster placements (*n* = 3), and case workers who serve youth in care (*n* = 3). Foster parents in Phase I were recruited through three channels: (a) community agencies that serve or license foster parents in Illinois, including the Illinois Department of Children and Family Services (DCFS) and contracted private foster care agencies; (b) targeted social-media outreach to foster parent and kinship caregiver groups; and (c) word-of-mouth referrals from agency partners and prior research participants. This mirrored the recruitment strategy used in Phases II and III to maximize comparability across phases. Purposive sampling was used to recruit caregivers varying in race and ethnicity, primary language (English or Spanish), caregiving role (licensed foster parent, kinship caregiver, or foster-to-adopt caregiver), and years of caregiving experience. Mental health providers and caseworkers were recruited through partnering child welfare agencies and community mental health clinics serving foster-involved youth in Illinois. Participants received a $50 digital gift card for participation.

During these 60-to-90-min, video-taped, semi-structured focus group interviews, participants were asked to examine a high-fidelity prototype of the FN + curricula. We elicited feedback about all aspects of the curricula and obtained feedback regarding the clarity of terminology and content, relevance of concepts, preferences for different types of activities or methods of skill demonstration (e.g., video, role play, scenario discussion, etc.). Following transcription and review of recorded sessions, study staff used an interpretive grounded theory approach to identify the highest priority areas for adapting materials.

### 2.2 Phase II. Pilot feasibility open trial

#### 2.2.1 Open pilot participants

For the ongoing phase II open pilot trial, we are recruiting 12 foster parents via word of mouth, social media, and community agencies that serve or license foster parents in the state of Illinois. Inclusion criteria are as follows: 1) living in Illinois, 2) age 18-years old or older, 2) caregiver of a youth in foster or kinship care currently under their care or adopted from foster or kinship care, 3) caregiver of a youth that has been under their care for at least 30 days within the past 6 months, 4) English or Spanish speaking, 5) primary caregiver, 6) foster parent has not participated in any other phase of the FN + development or aspect of this study, 7) foster parent has reliable internet connectivity and access to a device that can accommodate videoconferencing, and 8) ability to provide informed consent. All open trial procedures have been approved by the institution and Illinois Department of Children and Family Services (DCFS) Institutional Review Boards.

#### 2.2.2 Procedures

Following informed consent, participants completed a battery of demographic and self-report questionnaires (see section 2.2.4). These questionnaires include parent-reports of their child's mental health concerns, which are used in the first session to provide the participant with personalized feedback on their perceptions of their internalizing, externalizing, and executive functioning, to support customized resources and referrals. Participants then complete five 40–60-min semi-structured videoconference sessions led by family navigators. The family navigator team consists of three members with varying levels of clinical experience: one with no prior clinical experience, one with minimal experience (e.g., two years as a support practitioner), and one master's-level clinician who practiced as a licensed professional counselor for two years. Following completion of each session, participants provide verbal and written feedback. Data are also collected in a post-intervention assessment and a 3-month follow-up assessment. Participants are paid $20 for each assessment completed (baseline, post-intervention, 3-month) in the form of a digital gift card.

#### 2.2.3 FN + program

FN+ is a five-week, skills-based program designed to increase foster parent activation and confidence in managing their child's mental health needs. Family navigators meet with foster parents weekly via HIPAA-compliant videoconferencing for 45–75-min sessions, each organized around 2–4 core learning objectives and one key takeaway to reinforce skill application between sessions. In Session 1, foster parents are introduced to the structure and goals of FN+, meet their family navigator, and discuss their child's mental health symptoms, behavioral patterns, and strengths to inform individualized resources and referrals. The family navigator also reviews the parent's report of their child's mental health symptoms (see Measures below). Session 2 focuses on helping foster parents navigate state mental health systems, understand the value of evidence-based treatments, and problem-solve barriers to accessing care with family navigator support. In Session 3, foster parents learn about the purpose and goals of mental health treatment, build skills to collaborate effectively with providers, and develop strategies to address barriers to treatment engagement—even if services have not yet begun. Session 4 introduces the functions of behavior and the Antecedents, Behavior, and Consequences (ABCs) framework, enabling foster parents to identify patterns that contribute to emotional or behavioral difficulties and respond more effectively. In the final session, family navigators train foster parents to locate, evaluate, and implement high-quality digital mental health tools, supporting the use of symptom-specific online resources to enhance their child's care. Table 1 provides an overview of FN + program components.

**Table 1:** FN + program components.

**Session 1**	Understanding Your Child's Symptom Profile	Provide an overview of the program and help parents better understand their child's symptoms through idiographic assessment.
**Session 2**	Child Mental Health Treatment	Train parents to find and overcome barriers to accessing mental health services. Understand the importance of evidence-based treatment.
**Session 3**	Managing Your Child's Mental Health Treatment	Build parent capacity to understand the child's treatment goals, communicate with providers, and support treatment goals at home.
**Session 4**	Understanding the “why" Child Behaviors	Train parents understanding the functions of child behaviors focusing on antecedents, behavior, and consequences (ABCs)
**Session 5**	Using online mental health tools at home	Train parents to find, evaluate, and use online mental health tools

#### 2.2.4 Measures

*Feasibility and Acceptability.* Feasibility and acceptability are the primary outcomes for the open trial. Participants will complete a Post-session Questionnaire following each FN + session. This questionnaire was designed to assess the utility and relevance of session content and materials. In addition, the family navigator asks participants about the skills or resources the participant used the previous week. Parent attendance and drop-out will serve as indicators of program acceptability; recruitment feasibility will be measured as the ability to recruit the intended population. Following the 5-week intervention, participants will also complete the Client Satisfaction Questionnaire (CSQ-8) [27]. The CSQ-8 assesses participant satisfaction with the services received including the overall quality, their perception of whether the program met their needs, amount of help received, and the extent to which FN + helped them navigate their child's mental health needs more effectively. The CSQ-8 demonstrates good internal consistency, test-retest reliability, and is sensitive to treatment effects [29].

*Pilot of primary and secondary outcome measures.* We will also pilot the planned outcome measures for the RCT. The primary outcome measure, the Parent-Patient Activation Measure-Mental Health (P-PAM-MH) [28], will be used to assess foster parent activation in the child's mental health care. The P-PAM-MH is a 13-item measure designed to assess foster parent knowledge, skills, and capacity to manage the child's mental health and mental health care. The measure has demonstrated good test-retest reliability and convergent validity [28]. Secondary outcome measures assess barriers to treatment participation and the perceived change in capacity to manage youth mental health care. The Barriers to Treatment Participation Scale (BTPS) [30] was designed to assess parent's perceptions of barriers to mental health treatment engagement for their children. It consists of four subscales that measure, 1) stressors and obstacles that compete with treatment, 2) treatment demands and issues, 3) perceived relevance of treatment, and 4) relationship with the therapist. To measure Perceived Change, two items were developed for the present study that ask participants to rate, compared to before the intervention, their perceived ability to: 1) more accurately identify the antecedents of their child's behaviors; and 2) more effectively manage their child's difficult behavior at home.

*Parent report of child mental health symptoms.* In the first FN + session, family navigators provide the participants a report of their child's mental health symptoms based on the following questionnaires completed at the baseline assessment: 1) The Pediatric Symptom Checklist-17 [31] contains three subscales assessing symptoms associated with internalizing, externalizing and attention problems. Psychometric properties are strong [2,32,33]) The PROMIS Parent Proxy Pediatric Anxiety Short Form 8a [34] assesses symptoms of fear, anxious misery, hyperarousal, and somatic symptoms related to arousal children. It has demonstrated good internal consistency, test-retest reliability, and convergent validity, and 3) The PROMIS Parent Proxy Pediatric Depressive Symptoms Short Form 6a [34] assess negative mood and views of self, social cognition, and decreased positive affect and engagement in children. It has demonstrated good internal consistency, test-retest reliability, and convergent validity [34,35].

#### 2.2.5 Data analyses

We will examine the by-session and end of intervention feedback provided by participants. Given the small sample size and the primary feasibility focus, analyses will be restricted to descriptive statistics (Means, *SD*s) for primary and secondary outcome measures. To quantify the magnitude of observed changes, effect sizes (Cohen's *d*) will be calculated.

#### 2.2.6 Data safety monitoring board

An external Data Safety Monitoring Board (DSMB) was created to oversee participant safety in the open and randomized controlled trials. The five board members are experienced with foster youth and youth mental health needs, human participant protections, safety procedures, and mental health services. The DSMB will meet monthly to review all withdrawals, subject removals, and clinically elevated parent-reports of child behaviors, and actions taken in response. The DSMB will also review any serious adverse events, adverse events, and participant safter and make recommendations to the National Institute of Mental Health regarding continuation, modification or termination of the study.

## 3 Phase III. Pilot feasibility RCT

### 3.1 Design overview

Phase III includes a 5-week, single site pilot feasibility RCT of the FN + program as compared to a civic engagement attention control condition (CE). The attention control condition was designed to serve as a rigorous comparison group by providing foster parents with equivalent time, attention, knowledge, skills, and confidence-building opportunities as the treatment condition, but focused on youth civic engagement rather than mental health. By matching structure and intensity while differing in content, any observed differences in outcomes between groups can be attributed to the mental health–specific components of the FN + intervention. Primary and secondary outcomes are assessed after the 5-week intervention, and at 3-month follow-up. All study procedures are approved by the institutional and Illinois DCFS Institutional Review Boards. The trial is registered on clinical trials.gov (NCT06978959).

### 3.2 Specific aims and hypotheses

1. Examine the feasibility and acceptability of research procedures and interventions
2. Test the efficacy of FN+ in improving parental activation. We hypothesize that foster parents receiving the FN + intervention will demonstrate increased parental activation relative to those receiving the civic engagement attention control.
3. To examine foster parent perception of their capacity to access mental health services for their child, support their child's treatment goals at home, and more effectively manage their child's behavior. We hypothesize the foster parents receiving FN + will demonstrate improved capacity to manage youth mental health relative to the attention control condition.

### 3.3 Participants

Participants (*N* = 60) will be recruited from community agencies that serve or license foster parents in the state of Illinois, social media, and word of mouth. Inclusion criteria are the same as the open trial.

### 3.4 Randomization

Following eligibility determination, participants will be randomized 1:1 to either the intervention (FN+) or attention control condition (CE), stratifying for age of child. Three developmentally informed strata will be used: early childhood (5–9 years), middle childhood/early adolescence (10–13 years), and middle/late adolescence (14–17 years). These strata were selected to reflect clinically meaningful differences in mental health symptom presentation, predominant treatment modalities offered, age-related considerations related to care navigation and decisional autonomy. Stratified block randomization (block size of three) will be implemented within each age stratum, using a pre-generated allocation sequence held by a study team member not involved in enrollment, to maintain balance across the two conditions within strata.

### 3.5 Interventions

*Family Navigator Plus (FN+).* See section 2.2.3.

*Civic Engagement.* Civic engagement—activities intended to address public issues and strengthen communities—was selected as the attention control condition. It mirrors the structure and format of FN+, providing foster parents with equivalent time, attention, knowledge, skills, and confidence-building opportunities, but focuses on youth civic engagement rather than mental health. Session 1 introduces the program and family navigator, defines civic engagement within a developmental framework, and guides foster parents in discussing their child's developmental level, strengths, and opportunities for civic participation. Session 2 focuses on identifying developmentally appropriate civic activities and strategies to initiate and support youth involvement. Session 3 emphasizes goal setting, age-appropriate engagement strategies, collaboration with caseworkers, and structured problem-solving to address barriers to participation. Session 4 supports foster parents in setting developmentally appropriate civic engagement goals while strengthening active listening and validation skills to promote youth autonomy. The final session trains foster parents to locate, evaluate, and use high-quality online civic engagement resources to sustain ongoing involvement. Table 2 provides an overview of civic engagement program components.

**Table 2:** Civic engagement program components.

**Session 1**	Understanding Your Child's Developmental Stage	Provide an overview of typical child development and developmentally appropriate civic engagement behaviors
**Session 2**	Initiating Youth Civic Engagement	Discuss developmentally appropriate methods to promote youth civic engagement.
**Session 3**	Promoting Youth Civic Engagement	Build parent capacity to support developmentally appropriate civic engagement.
**Session 4**	Understanding the “why" of civic engagement	Describe the elements of civic engagement that support youth development.
**Session 5**	Using online tools at home	Train parents to find, evaluate, and use online tools to support civic engagement

### 3.6 Measures

We plan to use the same measures piloted in the open trial (see Section 2.2.4).

### 3.7 Monitoring of intervention integrity

Intervention integrity will be assessed by reviewing 10% of randomly selected audio recordings of FN+ and CE sessions. To increase adherence, family navigators will be provided with a list of allowed and disallowed interventions in each condition. They will also fill out and self-reported adherence checklist at the end of each session. Finally, all family navigators will meet weekly with the PIs to ensure fidelity to the interventions.

### 3.8 Statistical analysis

#### 3.8.1 Primary outcome

The primary outcome for this study is parent activation. We will examine change in foster parent activation on the P-PAM-MH from baseline to subsequent time points in the intervention, relative to the attention control condition. Foster parent activation will be measured using the P-PAM-MH. Data will be analyzed using two parallel analytic methods: (1) a 2 (Condition) x 3 (Time) repeated measures ANOVA, and (2) a corresponding 2 x 2 repeated measures ANCOVA (using Baseline as a covariate). The ANOVA model evaluates contrasts in actual outcomes, and the ANCOVA model evaluates adjusted mean contrasts, statistically equating groups on baseline status (thus correcting possible group differences at Baseline, and as is often the case, increasing statistical power using baseline as a covariate). We will conduct preliminary analyses to test for additional covariates such as level of education. For the ANOVA model, in addition to time (*df* = 2) and group by time effects (*df* = 2), pairwise contrasts of outcomes at the 3 times will be conducted on the time factor. In addition to overall treatment group effects, treatment groups will be contrasted on average P-PAM-MH within times, and on change between Baseline and the two post-baseline time points. For the ANCOVA models, groups will be contrasted on the adjusted mean average across all times, on adjusted means at each post-baseline time, and on the difference between the 2 post-baseline adjusted means.

#### 3.8.2 Secondary outcomes

The secondary outcomes entail both continuous and categorical variables. For the continuous variables (subscales and total score on BTPS, and sum of discrete types of barriers), the analytic models described above will be applied. In addition, corresponding overall multivariate tests treating the outcomes as a dependent variable vector will be conducted (RM MANOVA and MANCOVA). For the parent perception of capacity after intervention items, we will use t-tests to examine perceived change and to estimate effect. For categorical items on the BTPS we will use McNemar's test.

#### 3.8.3 Power analyses and sample size considerations

Effect sizes yielding 80% power were estimated based on a sample size of 60 subjects, and assuming a type 1 error rate of α = .05, and assuming a sphericity correction of ε = 0.75 for 2 *df* mixed ANOVA models, and an average correlation between all repeated measures of *r* = .5 for all models. Tests of the overall 2x3 mixed ANOVA interaction achieved 80% power at an f-ratio effect size of *f* = 0.184 (i.e., partial η2 = .0327), and interaction tests of contrasts of pairs of times had 80% power for effect size *f* = 0.184 (i.e., pη2 = .0327). ANCOVA models include baseline as a covariate and predict subsequent outcome variance. With respect to predictions at a single post-baseline assessment, 80% power is present to detect an effect size of partial *f* = 0.368 (i.e., pη2 = .119). Both detectable effect size estimates rely strongly on assumptions about error structure. The pilot data will provide new error structure information (within the context of the models above) that will improve future power estimates for a broader RCT. Although this study is not adequately powered to detect small effects, we will generate data sufficient to better inform future power analyses with respect to error structure and effect sizes.

Consistent with NIH guidance for R34 pilot and feasibility studies, the sample size was selected to evaluate feasibility, acceptability, recruitment procedures, and preliminary signals of effect, rather than to definitively test efficacy. Accordingly, the effect sizes reported above reflect the minimum effects detectable at the predetermined feasibility-based sample size, not the effects the current study is powered to confirm. Age stratification (5–9, 10–13, and 14–17 years) will be used to maintain balance on a clinically important developmental variable while preserving the overall sample size. Because the study is not powered to detect age-stratum-specific effects, age will be examined descriptively and included as a covariate in exploratory sensitivity analyses. Pilot data, including stratum-specific variance and correlation estimates, will be used to refine sample size estimates for the subsequent definitive RCT.

## 4 Discussion

This study seeks to address a critical and persistent gap in the mental health care of youth in foster care by targeting parent activation and barriers to treatment engagement. Given the high burden of psychiatric morbidity and the well-documented delays and disparities in accessing evidence-based treatment, interventions that strengthen caregivers’ capacity to recognize needs, navigate systems, and sustain engagement in care are urgently needed. By adapting a family navigation model to the unique structural constraints of child welfare and delivering it via telehealth to enhance accessibility, FN + seeks to reduce practical barriers while building durable caregiver skills. The phased development approach—including stakeholder-informed refinement, open and randomized-controlled trials—positions this study to generate preliminary evidence regarding feasibility, acceptability, and potential efficacy. If FN + demonstrates improvements in foster parent activation, it may represent a scalable, systems-aligned strategy to promote earlier intervention, reduce placement instability linked to unmanaged behavioral health needs, and ultimately mitigate long-term psychiatric risk among youth in care.

## Funding statement

This study was funded by 10.13039/100000025NIMH grant R34MH134941 (Risser and Boisseau, PIs). The funding agency was not involved in the study design, analysis and interpretation of data, writing of report, or the decision to submit this article for publication.

## CRediT authorship contribution statement

**Christina L. Boisseau:** Conceptualization, Funding acquisition, Investigation, Methodology, Project administration, Resources, Supervision, Writing – original draft. **Heather Risser:** Conceptualization, Funding acquisition, Investigation, Methodology, Project administration, Resources, Supervision, Writing – review & editing. **Miguel A. Herrera:** Writing – review & editing. **Clara Law:** Project administration, Writing – review & editing. **Zoran Martinovich:** Conceptualization, Investigation, Methodology, Writing – review & editing. **Spencer St. Jean:** Writing – review & editing. **Yufan Wang:** Writing – review & editing.

## Declaration of competing interest

The authors declare that they have no known competing financial interests or personal relationships that could have appeared to influence the work reported in this paper.

## Data Availability

No data was used for the research described in the article.
